# Effect of Long-Term Treatment with Fimasartan on Transient Focal Ischemia in Rat Brain

**DOI:** 10.1155/2015/295925

**Published:** 2015-09-13

**Authors:** Chi Kyung Kim, Xiu-Li Yang, Young-Ju Kim, In-Young Choi, Han-Gil Jeong, Hong-Kyun Park, Dohoung Kim, Tae Jung Kim, Hyunduk Jang, Sang-Bae Ko, Byung-Woo Yoon

**Affiliations:** ^1^Department of Neurology, Seoul National University Hospital, Seoul 03080, Republic of Korea; ^2^Biomedical Research Institute, Seoul National University Hospital, Seoul 03080, Republic of Korea; ^3^Neuroscience Research Institute, Seoul National University College of Medicine, Seoul 03087, Republic of Korea

## Abstract

Fimasartan is a newly developed angiotensin receptor blocker, which may have protective effects during myocardial infarction or atherosclerosis. In this context, we investigated the effects of long-term treatment with low-dose fimasartan on focal ischemia in rat brain. We induced focal ischemia in brain by transient intraluminal occlusion of middle cerebral artery (MCA) and administered low-dose (0.5 mg/kg) or regular doses (1 or 3 mg/kg) of fimasartan via intravenous routes. After the administration of low-dose (0.5 mg/kg) fimasartan, blood pressure did not decrease compared to the phosphate-buffered saline- (PBS-) control with MCA occlusion (MCAO) group. The infarct volume and ischemic cell death were reduced in the low-dose fimasartan-treated group (46 ± 41 mm^3^ for 0.5 mg/kg and 153 ± 47 mm^3^ for PBS-control with MCAO; *P* < 0.01) but not in the regular-dose groups. Low-dose fimasartan treatment improved functional recovery after ischemia and significantly decreased mortality. In our study, fimasartan reduced the degradation of I*κ*B and the formation of an inflammatory end-product, COX-2. As a result, the recruitment of inflammatory cells in the peri-infarct area decreased in fimasartan-treated group. We have demonstrated that long-term, low-dose fimasartan treatment improved outcomes after focal ischemia in the brain via a reduction of inflammation.

## 1. Introduction

Fimasartan (BR-A-657) is the ninth developed angiotensin receptor blocker (ARB) from Korea [1]. Fimasartan is a series of pyrimidin-4(3H)-one derivatives as losartan analogues, and fimasartan has about six hundredfold affinity for the angiotensin II receptor type 1 (AT1) compared to equivalent doses of losartan [2, 3]. In addition to greater potency, fimasartan is rapidly absorbed following oral administration, and the time to peak plasma concentration ranges 0.5–3 h with comparable safety profile [1]. Thus, it has been used as an antihypertensive drug in Korea since 2011 after approval by the Korean Food and Drug Administration. In addition to lowering blood pressure (BP), there are a few experimental studies suggesting fimasartan has protective effects during cardiovascular diseases. Fimasartan decreased infarct volume and improved functional outcomes during rat myocardial infarction [4], and it prevented the progression of atherosclerosis in injured vessels in a rabbit model [5]. However, the effect of fimasartan on focal ischemia in the brain has not been investigated.

Stroke is one of the leading causes of mortality worldwide including Korea and presents a disease burden to patients' families and society due to disability after stroke [6]. Despite these detrimental outcomes, stroke treatment has been limited to recanalization therapy, and there is no practical neuroprotective agent [7, 8]. Some ARBs demonstrated neuroprotective effects in animal models against ischemic stroke [9–11]. However, one of those ARBs (candesartan) did not demonstrate any beneficial effect for ischemic stroke patients when administered in the acute phase and may have even been harmful [12]. It has been postulated that this failure may originate from lowered BP in the candesartan treatment group because tissue hypoperfusion might accelerate ischemic damage in the acute phase after stroke. In some diseases, such as diabetic nephropathy in which the relative hypotension may promote the progression of the disease, the effects of low-dose ARBs have been studied [13, 14]. Furthermore, physicians usually choose antihypertensive drugs according to comorbidities of patients and adverse events of drugs, not by the outcomes of cardiovascular events related to hypertension. However, a recent retrospective cohort study showed that prestroke administration of ARBs might improve the outcomes of stroke [15]. In this context, we investigated the effects of long-term treatment with a low-dose novel ARB, fimasartan, on focal ischemia in the rat brain.

## 2. Materials and Methods

### 2.1. Animals and Experimental Groups

**Figure 1:**
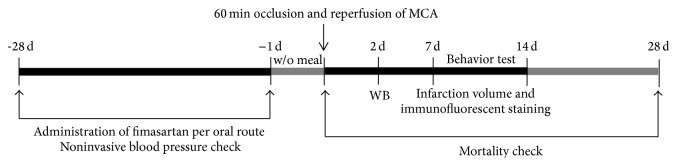
Schematic diagram of the study protocols. For Sprague-Dawley rats, low-dose (0.5 mg/kg) or regular doses (1 or 3 mg/kg) were administered for 4 weeks via an oral route before the induction of transient middle cerebral artery (MCA) occlusion. At 2 days, western blot (WB) analyses were performed. At 7 days immunofluorescent staining was performed, and infarct volume was measured. Behavior tests were performed for 14 days after induction of transient MCA occlusion, and mortality was censored at 28 days. Blood pressure was monitored during the pretreatment and follow-up period.

Male Sprague-Dawley rats (Koatech, Seoul, Republic of Korea), weighing between 200 and 220 g, were used in these experiments. All animal studies were performed according to the National Institutes of Health Guide for the Institutional Animal Care and Use Committee of the Biomedical Research Institute at Seoul National University Hospital. We administered fimasartan at a low-dose (0.5 mg/kg) and a regular dose (1 or 3 mg/kg) or phosphate-buffered saline (PBS) orally for 4 weeks once per day before the induction of ischemia and reperfusion in the middle cerebral artery (MCA). After induction of transient MCA occlusion (MCAO), we ceased the fimasartan administration to prevent BP reductions after ischemia (Figure 1).

### 2.2. Focal Ischemia-Reperfusion Model

Focal cerebral ischemia-reperfusion was induced with a minor modification of the endovascular internal carotid artery (ICA) suture method [16, 17]. After inhalation of 3% isoflurane in 30% oxygen and 70% air, the left common carotid artery (CCA) was exposed at its bifurcation using a midline cervical incision. The external carotid artery (ECA), ICA, and CCA were ligated using a 5-0 silk suture. The CCA was then transected, and a 5-0 nylon monofilament suture (with its tip rounded by heating) was inserted into the CCA. To occlude the origins of the MCA and proximal anterior cerebral artery, the suture was advanced into the ICA for a distance of 20 mm. The suture was secured in place using a ligature, and the wound was closed. The monofilament was removed 60 min after the occlusion. The animals were allowed food and water ad libitum. Rectal temperature was maintained at 37 ± 0.5°C using a thermistor-controlled heating blanket. Sham operations were performed to make a negative control group (*n* = 4 for Nissl staining and *n* = 2 for TTC staining).

### 2.3. Measurement of Infarct Volumes

After cardiac perfusion-fixation with 4% paraformaldehyde in 0.1 mol/L PBS, the brains were removed quickly and cut into 30 *μ*m thick coronal sections on a freezing microtome. Ten brain sections were mounted onto glass slides and processed for Nissl staining for measurement of infarct volumes, and to demonstrate infarct areas clearly, TTC (2,3,5-triphenyltetrazolium chloride) staining was performed (*n* = 3 for each group). The infarct volumes were measured using an image analysis program, ImageJ (National Institutes of Health, Bethesda, MD).

### 2.4. *In Situ* Labeling of DNA Fragmentation

Terminal deoxynucleotidyl transferase-mediated dUTP-biotin nick end labeling (TUNEL) was performed with the use of a commercially available kit as described previously [18]. Sections were incubated in a TdT-labeling reaction mixture for 90 min, colored with DAB solution, and counterstained with methyl green. A single axial section through the center of the ischemic lesion was analyzed. Eight sampling regions were placed along the periphery. TUNEL-positive cells were identified and counted. Total counts in these sampling regions were converted into cell densities for quantification and comparison between the treatment and control groups.

### 2.5. Behavioral Testing and Mortality Check

This test was a modified version of a test described by a previous study [19]. The limb-placing test was used to evaluate the outcome of recovery on postoperative days 1, 3, 7, and 14. The test assesses the sensorimotor integration of the forelimb and the hind limb by checking responses to tactile and proprioceptive stimulation. In the first task, the rat was suspended 10 cm over a table, and the forelimb stretch towards the table was observed. In the second test, the rat was positioned towards the table and its forelimbs were placed on the table. Next, the rats were placed along the table edge to check for lateral placement of the forelimb (third task). In the fourth task, the rat was again positioned towards the table with the hind limbs just over the table edge. Each hind limb was pulled down and gently stimulated by pushing towards the side of the table. The 4 tasks were scored in the following manner: normal performance, 0 points; incomplete performance, 1 point; no performance, 2 points. A total of 8 points indicated maximal neurological deficit, and 0 points indicated normal performance. The mortality was checked 28 days after induction of transient MCAO.

### 2.6. Measurement of Blood Pressures

The BP was recorded using a CODA Noninvasive Blood Pressure System (Kent Scientific Corporation, Torrington, CT). The BP is recorded by a band attached to the tail (homologated by Bland-Altman testing) [20]. This method is recommended by the American Heart Association as a measuring guide for laboratory animals [21]. Noninvasive BP monitoring was performed on days 28, 27, 25, 21, 14, and 7, just before MCAO induction. After MCAO, noninvasive BPs were measured on days 1, 3, and 7. Invasive BPs were obtained via the femoral artery once before ischemia (after fimasartan administration for 28 days).

### 2.7. Immunofluorescent Staining and Cell Quantification

Immunofluorescent staining of brain tissue was performed using cryopreserved 40 *μ*m coronal sections. Each section was incubated with 0.5% bovine serum albumin/0.3% Triton-X followed by 10% normal serum in PBS for 1 hour for blocking. Sections with a primary antibody were placed at 4°C for 16 hours. After washing, each section was subsequently incubated for 2 hours at room temperature with the fluorophore-conjugated secondary antibody. Monoclonal antibodies against MHC class II Ia (Ox6; Santa Cruz Biotech, Santa Cruz, CA) labeled activated microglia/macrophages. Stained cells were then examined under a confocal laser scanning biological microscope (LSM 410 META; Carl Zeiss, Jena, Germany).

Quantitative analysis of the positively stained cells was performed in the peri-infarct regions by two independent investigators (Hong-Kyun Park and Dohoung Kim) who were masked to the group allocations. To count activated microglia/macrophages, 16 high-power fields were taken from the sections through the center of the infarct lesion 7 days after transient MCAO. Total counts in the measured sections were converted into cell densities for comparison between fimasartan treatment and control groups according to our established protocol [22].

### 2.8. Western Blot Analysis

The rats were killed via decapitation, and the brains were immediately extracted 48 hours after the induction of transient MCAO. After the centrifugation of hemisphere homogenates, 50 *μ*g of protein was separated on a 10% sodium dodecyl sulfate-polyacrylamide gel electrophoresis gel and transferred to nitrocellulose membranes. These membranes were incubated in blocking buffer (5% skim milk in 50 mmol/L Tris PH 7.5, 0.15 mmol/L NaCl, and 0.05% Tween-20) and the blots were probed with antibodies recognizing I*κ*B (Cell Signaling Tech., Danvers, MA) and cyclooxygenase-2 (COX-2; BD Biosciences, Franklin Lakes, NJ). Immunoreactivity was visualized by enhanced chemiluminescence, and the relative optical densities were compared to the mean values of the control group.

### 2.9. Statistical Analysis

The values are presented as the means ± standard deviations. The data were analyzed with the nonparametric Mann-Whitney *U* test for unpaired samples between two groups, and the nonparametric Kruskal-Wallis *H* test was used for multiple groups. To compare each group after the Kruskal-Wallis *H* test, the Bonferroni correction was performed as post hoc test. A two-tailed value of *P* < 0.05 was considered significant. The survival analysis was performed according to the log-rank test. All statistical analyses were performed using SPSS 21.0 (SPSS Inc., Chicago, IL).

## 3. Results

### 3.1. Blood Pressure: Pretreatment and Follow-Up Period

**Figure 2:**
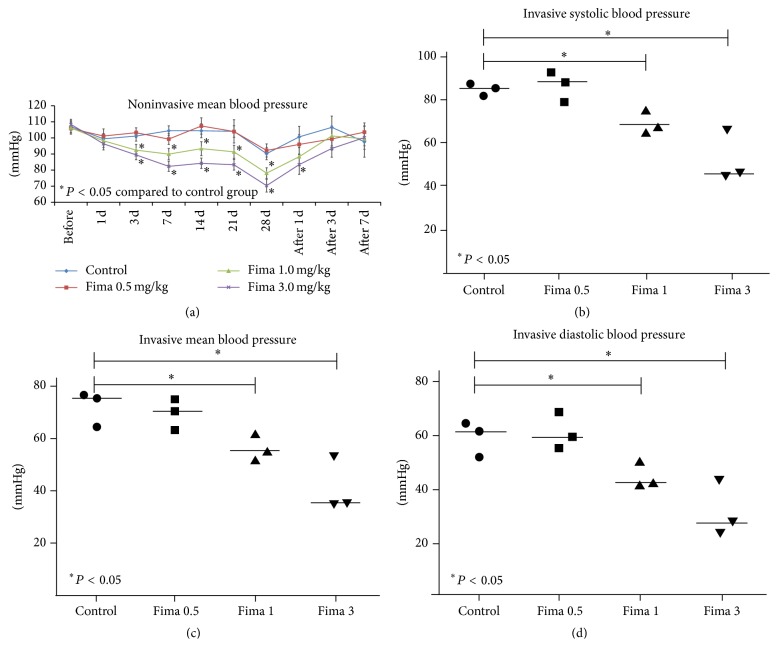
Blood pressure. (a) Mean blood pressures (MBPs) were decreased in the regular-dose fimasartan groups at 3 days after fimasartan administration. The MBPs in the low-dose fimasartan group were not different from the PBS-control with MCAO group (*n* = 9 each). (b) Invasive systolic blood pressures decreased in the regular-dose groups but did not decrease in the low-dose fimasartan group (*n* = 3 each). (c) Invasive MPBs in the low-dose fimasartan did not decrease compared to PBS-controls with MCAO (*n* = 3 each). (d) Invasive diastolic blood pressures decreased in the regular-dose groups but were not reduced in the low-dose group than PBS-control with MCAO group (*n* = 3 each).

The mean BPs decreased in the regular-dose fimasartan groups at 3 days, but the mean BPs in the low-dose fimasartan were not different from PBS-controls with MCAO via noninvasive monitoring (Figure 2(a)). Because of a one-day diet restriction prior to focal ischemia, the mean BPs in all groups were lower compared to the resting mean BPs in pretreatment period. With the single-time invasive monitoring just before focal ischemia, all BPs, including the systolic, diastolic, and mean, decreased in the regular-dose fimasartan group but did not decrease in the low-dose fimasartan group compared with PBS-controls with MCAO (Figures 2(b), 2(c), and 2(d)). After inducing focal ischemia, the mean BPs increased in all groups. The mean BPs in the regular-dose fimasartan groups returned to the level of the low-dose and control groups 3 days after inducing focal ischemia because we ceased fimasartan administration after ischemia to minimize the possible harmful effects of low BP.

### 3.2. Infarct Volume and Ischemic Cell Death

**Figure 3:**
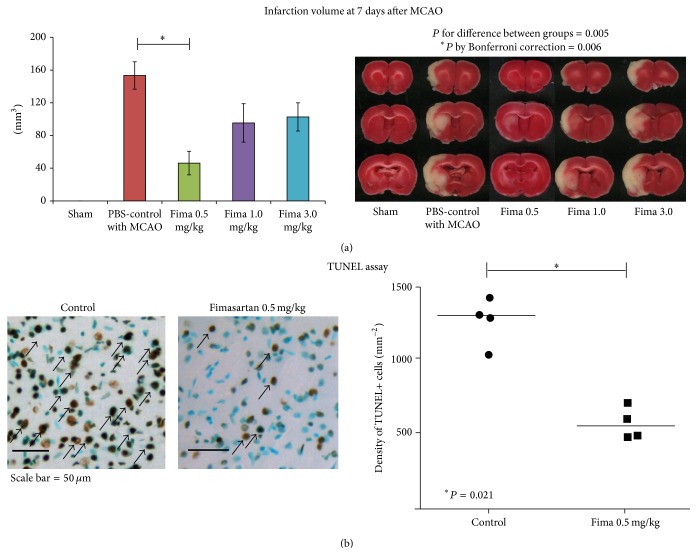
Measurements of infarct volume and ischemic cell death. (a) Sham operations did not make any infarct lesions in the brain (*n* = 6). The infarct volume in the low-dose fimasartan group (0.5 mg/kg, *n* = 8) decreased significantly compared to the PBS-control with MCAO group (*P* < 0.05 by Bonferroni correction, *n* = 8), but there was no difference between the regular-dose group and PBS-controls with MCAO (*P* for 1 mg/kg = 0.21 and *P* for 3 mg/kg = 0.12; *n* = 6 each). (b) Ischemic cell death, evaluated by TUNEL staining, decreased in the low-dose fimasartan group compared with the PBS-control with MCAO group (*P* < 0.05; *n* = 4 each). Representative TUNEL-positive cells (dark brown color) are indicated by black arrows, and TUNEL-negative cells are counterstained by methyl green (blue color). Scale bar = 50 *μ*m.

Sham operations did not make any infarct lesions in the brain (Figure 3(a)). The infarct volume decreased in the low-dose pretreatment groups compared to the PBS-control with MCAO group (46 ± 41 mm^3^ for 0.5 mg/kg and 153 ± 47 mm^3^ for control; *P* by Bonferroni correction between two groups < 0.01; Figure 3(a)). However, the infarct volumes in the regular-dose groups were not different from the PBS-control with MCAO group (95 ± 58 mm^3^ for 1 mg/kg and 103 ± 42 mm^3^ for 3 mg/kg; *P* by Bonferroni correction = 0.21 for 1 mg/kg and 0.12 for 3 mg/kg compared to the control group). Based on this result, we evaluated ischemic cell death, functional outcomes, mortality, and inflammatory changes between two groups (low-dose and control) to minimize the number of animals sacrificed. Ischemic cell deaths also decreased in the low-dose fimasartan group compared to the PBS-control with MCAO group as determined by TUNEL staining (578 ± 109 mm^−2^ for 0.5 mg/kg and 1270 ± 156 mm^−2^ for control group, *P* < 0.05; Figure 3(b)).

### 3.3. Functional Outcomes and Mortality

**Figure 4:**
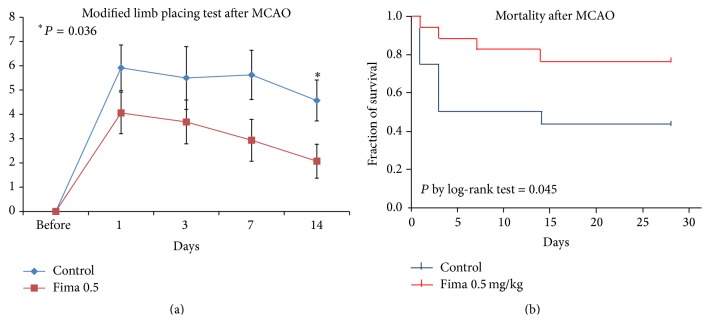
Functional recovery and mortality after focal cerebral ischemia. (a) Low-dose fimasartan improved functional recovery after transient focal ischemia compared to PBS-controls with MCAO. This improvement was prominent at 14 days after ischemia (*P* < 0.05; *n* = 17 for 0.5 mg/kg and *n* = 12 for control). (b) The mortality rate decreased significantly in the low-dose fimasartan group compared with PBS-controls with MCAO (*P* by log-rank test < 0.05; *n* = 18 each).

Low-dose fimasartan improved functional recovery after focal ischemia compared to the PBS-control with MCAO group. There was a trend for improved functional scores in the low-dose fimasartan group, and the functional recovery was prominent at 14 days after ischemia (2 ± 2 points for 0.5 mg/kg and 5 ± 3 points for control, *P* < 0.05; Figure 4(a)). The mortality rate decreased significantly in the low-dose fimasartan group compared with the PBS-control with MCAO (*P* by log-rank test < 0.05; Figure 4(b)).

### 3.4. Inflammatory Cell Recruitment and Inflammatory Markers

**Figure 5:**
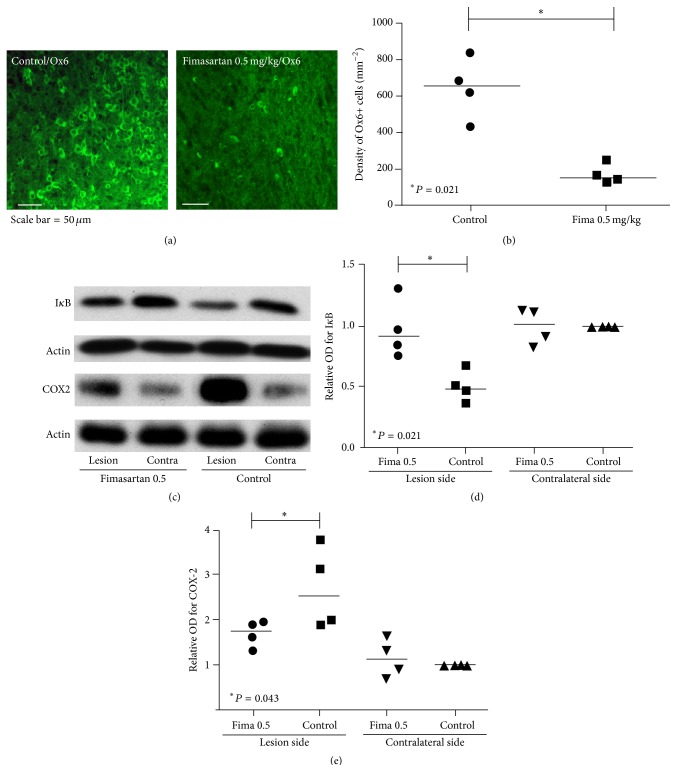
Immunofluorescent staining of inflammatory cells and western blot analyses. (a) Ox6-stained microglia/macrophages (green) were less frequently found in the periphery of the lesion, and the density in the fimasartan-treated group was lower than the PBS-control with MCAO group (*n* = 4 each). Scale bar = 50 *μ*m. (b) According to the quantitative analysis, the fimasartan-injected group exhibited a lower number of Ox6+ cells (*P* < 0.05) compared to the PBS-control with MCAO group. (c) At 48 hours after ischemia induction, the density of I*κ*B increased and cyclooxygenase-2 (COX-2) decreased in the fimasartan-treated group compared with the PBS-control with MCAO group (*n* = 4 each). (d) In the quantitative analyses, the degradation of I*κ*B was suppressed in the low-dose fimasartan-treated group in the lesion and was comparable to the contralateral brain in the PBS-control with MCAO group compared with lesion side in the PBS-control with MCAO group (*P* < 0.05). (e) The density of COX-2 in the infarcted hemisphere was 1.6-fold higher in the PBS-control with MCAO group compared to the fimasartan-treated group (*P* < 0.05).

Ox6-stained microglia/macrophages were less frequently found in the periphery around the lesion, and the density in the low-dose fimasartan-treated group was lower than the PBS-control with MCAO group (Figure 5(a)). According to quantitative analysis, the fimasartan-treated group exhibited a lower number of Ox6-positive cells (169 ± 54 versus 647 ± 167 cells/mm^2^) than the PBS-control with MCAO group (Figure 5(b)).

After ischemia, I*κ*B was degraded on the lesion hemisphere compared to the contralateral brain in PBS-control with MCAO animals (relative optical density on the lesion side for I*κ*B = 0.5 ± 0.1). In the low-dose fimasartan-treated group, the degradation of I*κ*B was suppressed to the level of the contralateral brain in control group (*P* < 0.05; Figures 5(c) and 5(d)). The density of COX-2, the inflammatory end-product after ischemia, in the infarcted hemisphere was 1.6-fold higher in the control group than the fimasartan-treated group (*P* < 0.05; Figures 5(c) and 5(e)).

## 4. Discussion

Fimasartan improved the outcomes after ischemic stroke in the experimental model and decreased inflammation related to ischemic damage. Inflammation is an important process that elicits acute damage after brain ischemia [23]. Compared to other pathologic processes after brain ischemia, inflammation is a potent reaction associated with reactive oxygen species, necrosis, apoptosis, and tissue remodeling. Moreover, it is closely related to various cell types such as microglia/macrophages, endothelial cells, neurons, and glial cells. In a recent* in vitro* study, fimasartan decreased inflammatory reactions in macrophages by reducing a proinflammatory transcription factor, NF-*κ*B, and inflammatory end-products such as iNOS [24]. This effect of fimasartan may have originated from the blocking of angiotensin II, which has been known to provoke inflammation through AT1 receptor [25]. This kind of anti-inflammatory effect by ARBs was also observed in the brain. Candesartan suppressed the expression of cytokine mRNA in LPS-induced inflammatory brain tissue [26]. In our study, fimasartan reduced the degradation of I*κ*B, which is a marker for NF-*κ*B activation, and the formation of an inflammatory end-product, COX-2. As a result, the recruitment of inflammatory cells in the peri-infarct area decreased after long-term fimasartan treatment.

Some other ARBs have shown neuroprotective effects against cerebral ischemia in hypertensive animals [27, 28]. The protective effects focused on the reversal of chronic vascular damage, which was induced by high BPs. In addition to this BP lowering effect, some investigators thought that ARBs might have direct protective effects against focal cerebral ischemia. To evaluate this, a few ARBs were investigated in normotensive animals. Irbesartan which was injected intracerebroventricularly decreased infarct volume, apoptosis, and inflammation independent of the systemic effects [9]. According to this observation, candesartan, which was administered via an intravenous or oral route, decreased infarct volume and improved functional outcomes after brain ischemia in normotensive rats [11, 29]. The experiments that delivered candesartan systemically were mainly focused on posttreatment after ischemia and regular doses of ARBs, which decreased BP. However, in a recent clinical trial, acute treatment with a regular dose of candesartan was not beneficial for ischemic stroke patients likely due to tissue hypoperfusion [12]. Similar to this human trial, we observed that low-dose fimasartan was superior to the regular doses. To reveal the protective effect of fimasartan independent of the BP lowering effects, we administered low-dose fimasartan to normotensive animals for a long period before inducing ischemia.

Low-dose ARB treatments have been evaluated in some disease models [30, 31]. Unlike myocardial infarction where high BP is directly harmful to ischemic tissue due to the increased work load of the heart, the brain and kidney are more vulnerable to relative hypotension in ischemic states than the heart. For diabetic nephropathy, the beneficial effects of low-dose ARBs have already been reported [13, 14], and there are ongoing clinical trials investigating the effects of low-dose fimasartan on diabetic nephropathy. Without adequate oxygen and glucose, neurons could not survive for more than a few minutes [32]. In this context, lowering the BP during the acute period after cerebral infarction should be applied cautiously. Low-dose ARB treatment might be considered as a neuroprotective treatment after cerebral infarction, and fimasartan is a good candidate for low-dose treatment because it has highly potent affinity for AT1 compared to other ARBs.

The present experiment had a few limitations. First, the protective effects of fimasartan on focal brain ischemia may not be exclusively caused by the anti-inflammatory effects of fimasartan. According to our observations, reducing inflammation could be one of the important beneficial processes of fimasartan, but other pathologic ways after cerebral ischemia such as apoptosis or disrupted autoregulation might be related to the effects of fimasartan. Second, to evaluate the protective effects of fimasartan independent of its BP lowering effects, our experiment was performed in normotensive animals. However, because fimasartan is mainly used as an antihypertensive drug, the neuroprotective effects of regular doses of fimasartan after cerebral ischemia in hypertensive animals should be investigated in future experiments.

## 5. Conclusion

Taken together, we have demonstrated that low-dose, long-term fimasartan treatment improved outcomes after focal brain ischemia via inflammation reduction. Fimasartan is already being used to treat hypertension in the daily practice of some countries because it has high potency and tolerable safety [1]. From our translational approach with fimasartan, we found a novel and safe therapeutic candidate to protect brain tissue after ischemia. After considering the lessons from experimental and clinical studies with other ARBs [12, 30], we applied an adequate dose of fimasartan so that its pleiotropic effect (independent of BP lowering) could be maximized. We believe that this protective effect of fimasartan will be investigated in human studies for the treatment of ischemic stroke in the near future.
